# Prevalence and correlates of depressive symptoms among adults living with HIV in rural Kilifi, Kenya

**DOI:** 10.1186/s12888-019-2339-5

**Published:** 2019-11-01

**Authors:** Moses K. Nyongesa, Paul Mwangi, Stanley W. Wanjala, Agnes M. Mutua, Charles R. J. C. Newton, Amina Abubakar

**Affiliations:** 1 Centre for Geographic Medicine Research-Coast, KEMRI, Box 230, Kilifi, Kenya; 2 Department of Social Sciences, Pwani University, Kilifi, Kenya; 3 Department of Public Health, Pwani University, Kilifi, Kenya; 4 Department of Psychiatry, University of Oxford, Oxford, UK; 5 Institute for Human Development, Aga Khan University, Nairobi, Kenya

**Keywords:** Depressive symptoms, Prevalence, HIV/AIDS, cART, Correlates, Kenya

## Abstract

**Background:**

Published research on depression among people living with HIV/AIDS (PLWHA) from Africa is increasing, but data from Kenya remains scarce. This cross-sectional study measured the prevalence and correlates of depressive symptoms among PLWHA in rural Kilifi, on the Kenyan coast.

**Methods:**

Between February and April 2018, we consecutively recruited and interviewed 450 adults living with HIV and on combination antiretroviral therapy (cART). Depressive symptoms were assessed with the 9-item Patient Health Questionnaire (PHQ-9), with a positive depression screen defined as PHQ-9 score ≥ 10. Measures of psychosocial, health, and treatment characteristics were also administered.

**Results:**

The overall prevalence of depressive symptoms was *13.8% (95% Confidence Interval (95%CI): 10.9, 17.3)*. Multivariable logistic regression analysis identified current comorbid chronic illness *(adjusted Odds Ratio (aOR) 5.72, 95% CI: 2.28, 14.34; p < 0.001)*, cART regimen *(aOR 6.93, 95%CI: 2.34, 20.49; p < 0.001)*, perceived HIV-related stigma *(aOR 1.10, 95%CI: 1.05, 1.14, p < 0.001)* and difficulties accessing HIV care and treatment services *(aOR 2.37, 95%CI: 1.14, 4.91; p = 0.02)* as correlates of depressive symptoms.

**Conclusion:**

The prevalence of depressive symptoms among adults living with HIV on the Kenyan coast is high. Those at high risk for elevated depressive symptoms (e.g., with comorbid chronic illnesses, on second-line cART, experiencing perceived HIV-stigma or with problems accessing HIV care) may benefit from early identification, treatment or referral, which requires integration of mental health programmes into HIV primary care.

## Introduction

Depression remains a common mental disorder [1] and is projected to be the leading cause of disease burden worldwide by the year 2030 [2]. This neuropsychiatric disorder occurs two to three times higher in people living with HIV/AIDS (PLWHA) than in the general population [3, 4]. In low resourced settings of sub-Saharan Africa (SSA), where HIV/AIDS burden is enormous [5], prevalence of depressive symptoms is high; ranging from 14 to 32% in PLWHA on combination antiretroviral therapy (cART), and between 9 and 31% in mixed/untreated groups [4]. In Kenya, studies conducted among PLWHA have reported prevalence of major depressive disorder as 32% [6] and other depressive disorders as 15% [7].

Depression has deleterious consequences on the wellbeing and overall health of PLWHA. It may lead to alteration of economic productivity [8], reduced cART adherence [9], and poor quality of life [10]. Studies from SSA reported that depression is associated with poorer overall health status [11], increases the risk for suicidality [12], leads to faster disease progression and increased mortality [8].

Two recent studies have systematically reviewed the literature on correlates of depression among PLWHA within the SSA setting. One focused broadly on studies conducted within SSA [4], while the other specifically targeted studies conducted in Ethiopia [13]. Only one study [14] in the former review by Bernard et al. [4] contained data from Kenya, and the authors combined this data with data from Tanzania and Namibia in their analyses. From the reviewed studies, sociodemographic factors such as increasing age, not being married, low levels of education, unemployment, and lower income were significant correlates of depressive symptoms in PLWHA on cART. Psychosocial factors such as perceived HIV-related stigma and poor social support also consistently correlated with depressive symptoms. Inconsistent findings are reported on gender and cluster of differentiation 4 (CD4) cell count as correlates of depressive symptoms in both reviews.

Depression is a common neuropsychiatric disorder in PLWHA but has been neglected in SSA for a long time [8]. Noteworthy is that since 2010, the number of published reports on depression among PLWHA in Africa is increasing [4]. However, such research from Kenya, particularly the coastal region, remains limited. The only studies we are aware of, that have extensively looked into depression among PLWHA at the Kenyan coast, are those by Secor et al. [7] and Korhonen et al. [15], but these studies, focussed on key population of men who have sex with men. The present study, therefore, aimed to determine the prevalence of depressive symptoms and its correlates among adults receiving cART in Kilifi, coast of Kenya.

## Methods

### Study setting

This cross-sectional study was conducted in Kilifi County – coastal Kenya, between February and April 2018, through the Centre for Geographic Medicine Research (CGMR). Kilifi County consists mostly of a rural population. Majority of its residents are poor (71.4% live below poverty line), lack adequate formal education (only 7.1% have attained secondary education) and earn a living mainly through subsistence farming or fishing [16, 17]. Adult HIV prevalence is estimated at 5%, almost near the national average prevalence of 6% [18]. The socio-economic disparities in this setting may have a role to play in HIV infection and prognostic patterns. A public hospital – Kilifi County Hospital – from where participants were recruited, has a specialized HIV care and treatment clinic that was established in 2003. This HIV clinic provides comprehensive care services (including medical reviews, counselling, family planning, nutritional care, and antiretroviral dispensary) to PLWHA and serves as a research facility. About 60 patients are seen daily. To date, the clinic has enrolled over 9000 patients of all ages.

### Study participants

This work is part of data collected from a larger project looking at different outcomes in adults living with HIV, including health-related quality of life and mental health. Participants were patients attending an HIV care and treatment clinic at Kilifi County Hospital. To be included in the study, participants had to be adults, 18 to 60 years old, with confirmed HIV positive status, on cART, and providing consent for participation. We excluded participants who did not meet the inclusion criteria above. We did not include elderly individuals living with HIV (above 60 years) because of the increased likelihood of illnesses associated with advancing age, that may have an impact on the quality of life [19]. Eligible participants were also excluded if they could not comprehend and/or communicate in the national language, Kiswahili, which was used during the administration of all study instruments.

### Sampling and sample size estimation

Selection was done through consecutive sampling of eligible patients on arrival at the clinic until the required sample size was achieved. Sample size calculation was determined using the single population proportion formula. With precision set at 3.5% around a previously reported prevalence of 15.5% for depressive symptoms among adults living with HIV [20], a sample of 410 participants or more would give reliable prevalence estimates. To cater for a 10% non-response or systematic missing of data, non-contact, or other factors that tend to reduce the final sample size, we considered a sample of 450 as sufficient. This sample is > 95% powered to detect a proportion of ≥0.10 given a null proportion of 0.05 at 5% level of significance.

### Data collection procedures

For all participants, study instruments were interviewer-administered via android tablets, in the same order, and under the same administration environment. Research assistants received a 4-day training in research ethics and good interviewing techniques (with role plays) and were taught how to administer the tablet-based questionnaires. Questionnaire administration was done in a private and quiet room within the clinic setting, and the entire interview session lasted between 30 to 45 min.

#### Measures

##### Depressive symptoms

The 9-item Patient Health Questionnaire (PHQ-9) [21] was administered as a measure of depressive symptoms. This was preferred as it has been considered a reliable depression screening tool when used with PLWHA in Kenya [22], Uganda [23], and South Africa [23]. Also, recent guidelines from Kenya’s Ministry of Health [24] recommending screening for depressive symptoms with PHQ-9 in the routine care of patients on cART informed the choice of this measure. All the 9 PHQ items are rated on a Likert scale of “0” (not at all) to “3” (nearly every day), with the scores summated to derive a total score that ranges from 0 to 27. In terms of severity of depressive symptoms, scores of 5–9 points, 10–14 points, 15–19 points, and 20–27 points indicate, respectively, mild, moderate, moderately severe, and severe levels of depressive symptoms. Consistent with previous studies from SSA [23, 25, 26], a cut-off score of ≥10 was used in the present study to define a positive depression screen in the respondents. In SSA setting and with the adult HIV-population, this cut-off value provides the best combination of sensitivity (91.7%) and specificity (89.0%) [23]. PHQ-9 has previously been found to have good internal consistency (Cronbach’s alpha = 0.78) and acceptable test-retest reliability (intra-class correlation coefficient [ICC] = 0.59) when used among PLWHA in Kenya [22]. In this study, PHQ-9 internal consistency alpha was 0.81 (95% confidence interval [95% CI]: 0.78, 0.84) with a test-retest reliability of 0.78 (95% CI: 0.63, 0.87).

### Sociodemographic and asset index items

A researcher-developed sociodemographic questionnaire was used to capture participant’s age, sex, marital status, educational level, employment status, and people with whom they were living with. Additionally, an asset index form previously used in this setting [10] was used to gather information on disposable assets owned by participants as an indicator of their socioeconomic status.

### Health-related characteristics

During the face-to-face interviews, participants self-reported about current history of smoking, khat or alcohol use, and presence of any current opportunistic infection or chronic illness (that they were made aware of by their clinician) using “0” (no) or “1” (yes) response options.

### Treatment-related characteristics

In the interviews, participants were also asked about disclosure (if they had disclosed their seropositive status or not), their satisfaction with the current level of care (satisfied or not) and their perception on clinic accessibility (if current HIV-clinic was accessible or not). In responding to the latter item, participants were asked to consider the distance between where they were living and the location of the HIV-clinic.

### Clinical information

A clinical record form was used to extract participant data from the clinic’s medical record database on: dates of HIV-diagnosis and cART initiation, most recent cART regimen, World Health Organization (WHO) clinical staging, viral load, CD4 cell count, height and weight (for Body Mass Index [BMI]) calculation). We used patient-unique clinic numbers to access the medical records of our study participants. All participants gave informed consent for this clinical information to be retrieved from their clinic medical records.

### HIV-related stigma

The 12-item HIV stigma scale **[**27**]** was used to assess patient perceived HIV-related stigma under four dimensions of i) personalised stigma; ii) disclosure concerns; iii) negative self-image; and iv) concerns with public attitudes. Items on this scale are rated as “1” (strongly disagree), “2” (disagree) “3” (agree) and “4” (strongly agree). A total score derived from summated item scores ranges between 12 and 48. Higher scores indicate a greater level of perceived HIV-related stigma. In its initial validation, Cronbach’s alpha was > 0.7 [27]. In the present study Cronbach’s alpha was 0.81 (95% CI: 0.78, 0.83).

### Statistical analysis

Frequencies (with percentages) and means (with standard deviations) were used to describe sample characteristics. Departure from linearity in continuous variables was checked through visual inspection of the frequency histogram with normal overlay graph. Prevalence was presented as percentages. Univariate logistic regression analysis was used to assess the crude association between sample characteristics and positive depression screen (PHQ-9 cut-off score ≥ 10). Multivariable logistic regression analysis was used to investigate factors independently associated with a positive depression screen. Since there is no universal consensus in selecting predictor variables, we included all variables with *p* ≤ 0.20 from univariate logistic regression analysis in multivariable analysis. Bursac et al. [28] suggest that *p*-values less than 0.25 in univariate logistic regression may indicate some reasonable association with the outcome. Backward stepwise logistic regression models were fit in the multivariable model building process, removing all variables with *p* > 0.05 (one at a time). Age and sex were included regardless of the *p*-value as these have been reported to be associated with depressive symptoms in other studies from SSA [4, 6, 13, 14]. Regression diagnostics for correlation of covariates were performed, with no collinearity problems identified. For the final model, Hosmer-Lemeshow goodness of fit statistic (*p*-value > 0.05) was considered a well-fitting logistic regression model. All analyses were conducted in STATA (V.14.0) statistical software package [29] with *p* < 0.05 considered statistically significant for all tests of hypothesis.

## Results

### Sample characteristics

In total, 512 adult patients attending an HIV care and treatment clinic at Kilifi County Hospital were approached to participate in this study. Forty-four patients declined to participate after the study was explained to them in details. Eighteen patients were excluded because they were over 60 years (*n* = 11) or were unable to comprehend and/or communicate in Kiswahili (*n* = 7). Consequently, 450 patients, hereafter referred to as study participants, were consecutively recruited.

Table 1 presents sample characteristics aggregated by depressive symptom. Overall, the mean age of the participants was 42.7 (SD = 9.7) years. Most study participants were female (79.1%), had attained primary level education (53.1%), were unemployed (59.8%) and lived with a family member (82.4%). Only 12.7% of study participants were never married. A small proportion of study participants currently smoked (9.8%), used khat (4.4%) and were taking alcohol (11.4%). Chronic illnesses comorbid with HIV was present in 8.2% of the study participants, hypertension being the commonest (*n* = 20). The mean BMI of study participants was within the normal range (mean [SD] = 22.4 [4.8]). Most study participants were on first-line cART regimen (95.3%), and in stage 1 of the WHO clinical staging (93.7%). Only 6.0% of study participants had not disclosed their seropositive status. Less than a quarter of study participants (18.4%) had an opportunistic infection at the time of interview. Most participants found the HIV care and treatment clinic easily accessible (86.2%) and were generally satisfied with the current level of care they were receiving (95.8%).

**Table 1:** Participant characteristics by depressive symptoms and associated crude odd ratios

Sample Characteristic	Total sample	Positive depression screen		Unadjusted ORs	
		No	Yes*	ORs (95% CI)	*p*-value ^#^
Sociodemographic characteristics					
Age – years, mean (SD)	42.7 (9.7)	42.9 (9.8)	41.6 (9.4)	0.99 (0.96, 1.01)	0.34
Sex					
Female	356 (79.1)	304 (78.4)	52 (83.9)	Ref	
Male	94 (20.9)	84 (21.7)	10 (16.1)	0.70 (0.34, 1.43)	0.32
Marital status					
*Married/cohabiting*	196 (43.6)	177 (45.6)	19 (30.7)	Ref	
*Separated/Divorced/Widowed*	197 (43.8)	160 (41.2)	37 (59.7)	2.15 (1.19, 3.90)	0.01
*Never married*	57 (12.7)	51 (13.1)	6 (9.7)	1.10 (0.42, 2.89)	0.85
Education					
*Tertiary*	22 (4.9)	21 (5.4)	1 (1.6)	Ref	
*Secondary*	66 (14.7)	55 (14.2)	11 (17.7)	4.20 (0.51, 34.57)	0.18
*Primary*	239 (53.1)	208 (53.6)	31 (50.0)	3.13 (0.41, 24.10)	0.27
*None*	123 (27.3)	104 (26.8)	19 (30.7)	3.84 (0.49, 30.25)	0.20
Employment					
*Formally employed*	53 (11.8)	47 (12.1)	6 (9.7)	Ref	
*Self-employed*	117 (26.0)	101 (26.0)	16 (25.8)	1.24 (0.46, 3.37)	0.67
*Other*	11 (2.4)	10 (2.6)	1 (1.6)	0.78 (0.08, 7.24)	0.83
*Unemployed (including students)*	269 (59.8)	230 (59.3)	39 (62.9)	1.33 (0.53, 3.32)	0.54
Currently living with					
*Family*	371 (82.4)	320 (82.5)	51 (82.3)	Ref	
*Relative/friend*	10 (2.2)	7 (1.8)	3 (4.8)	2.69 (0.67, 10.74)	0.16
*Alone*	69 (15.3)	61 (15.7)	8 (12.9)	0.82 (0.37, 1.82)	0.63
Asset index score ^a^ – mean (SD)	1.2 (1.4)	1.3 (1.4)	1.1 (1.2)	0.89 (0.72, 1.10)	0.27
Perceived HIV-stigma score ^b^ – mean (SD)	28.4 (7.7)	27.7 (7.4)	32.9 (8.0)	1.09 (1.05, 1.13)	< 0.001
Health characteristics					
Currently smoking, OM = 1					
*No*	405 (90.2)	351 (90.7)	54 (87.1)	Ref	
*Yes*	44 (9.8)	36 (9.3)	8 (12.9)	1.44 (0.64, 3.27)	0.38
Current use of khat					
*No*	430 (95.6)	369 (95.1)	61 (98.4)	Ref	
*Yes*	20 (4.4)	19 (4.9)	1 (1.6)	0.32 (0.04, 2.42)	0.27
Current alcohol use, OM = 1					
*No*	398 (88.6)	346 (89.4)	52 (83.9)	Ref	
*Yes*	51 (11.4)	41 (10.6)	10 (16.1)	1.62 (0.77, 3.44)	0.21
Any current chronic illness					
*No*	413 (91.8)	363 (93.6)	50 (80.7)	Ref	
*Yes* ^*$*^	37 (8.2)	25 (6.4)	12 (19.4)	3.48 (1.65, 7.37)	< 0.001
Clinical characteristics					
BMI – kg/m^2^, mean (SD), OM = 4	22.4 (4.8)	22.5 (4.8)	21.5 (4.6)	0.95 (0.89, 1.01)	0.11
cART regimen, OM = 4					
*First line*	425 (95.3)	372 (96.6)	53 (86.9)	Ref	
*Second line* ^*1*^	21 (4.7)	13 (3.4)	8 (13.1)	4.32 (1.71, 10.91)	< 0.001
Viral load, OM = 145					
*≤ 1000 copies/mL*	265 (86.9)	232 (87.9)	33 (80.5)	Ref	
*> 1000 copies/mL*	40 (13.1)	32 (12.1)	8 (19.5)	1.76 (0.75, 4.14)	0.20
WHO clinical stage, OM = 5					
*Stage 1*	417 (93.7)	360 (93.8)	57 (93.4)	Ref	
*Stage 2*	22 (4.9)	18 (4.7)	4 (6.6)	1.40 (0.46, 4.30)	0.55
*Stage 3*	3 (0.7)	3 (0.8)	0 (0.0)	–	–
*Stage 4*	3 (0.7)	3 (0.8)	0 (0.0)	–	–
Months since HIV diagnosis – mean (SD)	96.8 (50.2)	95.4 (50.7)	105.5 (46.1)	1.00 (1.00, 1.01)	0.14
Months since cART initiation – mean (SD)	81.9 (47.1)	81.1 (47.4)	86.7 (45.2)	1.00 (1.00, 1.01)	0.38
Treatment characteristics					
HIV status disclosure					
*Yes*	423 (94.0)	364 (93.8)	59 (95.2)	Ref	
*No*	27 (6.0)	24 (6.2)	3 (4.8)	0.77 (0.23, 2.64)	0.68
Any current opportunistic infection					
*No*	367 (81.6)	323 (83.2)	44 (71.0)	Ref	
*Yes*	83 (18.4)	65 (16.8)	18 (29.0)	2.03 (1.10, 3.74)	0.02
Clinic accessibility, OM = 2					
*Yes*	386 (86.2)	340 (88.1)	46 (74.2)	Ref	
*No*	62 (13.8)	46 (11.9)	16 (25.8)	2.57 (1.35, 4.91)	< 0.001
Satisfaction with current care, OM = 1					
*Yes*	430 (95.8)	371 (95.9)	59 (95.2)	Ref	
*No*	19 (4.2)	16 (4.1)	3 (4.8)	1.18 (0.33, 4.17)	0.80

Notes: * cut-off score ≥ 10 on PHQ-9 # based on the Wald’s test $ one or more chronic illness in addition to HIV. Hypertension (54.1%), diabetes and cancer (8.1% each), kidney disease (2.7%), others (27.0%). 1 - all the participants were initially started on first line cART but at the time of data collection, the regimen had changed to second line. *ORs* odd ratios, *SD* standard deviation, *Ref* reference category, *OM* observation with missing value, *a* score range = 0 to 7, *b* score range = 12 to 48, *WHO* world health organization, *cART* combination antiretroviral therapy, *BMI* body mass indexNotes: *cut-off score ≥ 10 on PHQ-9 # based on the Wald’s test $ one or more chronic illness in addition to HIV. Hypertension (54.1%), diabetes and cancer (8.1% each), kidney disease (2.7%), others (27.0%). 1 - all the participants were initially started on first line cART but at the time of data collection, the regimen had changed to second line. *ORs* odd ratios, *SD* standard deviation, *Ref* reference category, *OM* observation with missing value, *a* score range = 0 to 7, *b* score range = 12 to 48, *WHO* world health organization, *cART* combination antiretroviral therapy, *BMI* body mass index

### Prevalence of depressive symptoms and severity

Using PHQ-9 cut-off score of ≥10, which has been shown to maximize on sensitivity and specificity for depression screening [23]; the overall prevalence of depressive symptoms was 13.8% in this sample. In terms of severity of depressive symptoms in the sample, 23.8% had mild, 9.6% had moderate, 3.6% had moderately severe, and 0.7% had severe depressive symptoms (Table 2).

**Table 2:** Prevalence of depressive symptoms

	Distribution (n out of *N* = 450)	Prevalence (95% CI)
Severity of depressive symptoms		
Mild	107	23.8 (20.1, 27.9)
Moderate	43	9.6 (7.2, 12.7)
Moderately severe	16	3.6 (2.2, 5.7)
Severe	3	0.7 (0.2, 2.1)
Positive depression screen (cut-off score ≥ 10)		
Yes	62	13.8 (10.9, 17.3)

Notes: 95% CI - 95% confidence interval

### Factors associated with depressive symptoms among adults living with HIV from coastal Kenya

Tables 1 and 3 present the results following univariate and multivariable logistic regression analyses for factors associated with positive depression screen among adults receiving cART in Kilifi. Even though viral load was associated with positive depression screen at *p = 0.20* in univariate analysis (Table 1), we did not include this variable in the multivariable logistic regression model because many patients had missing values (*n* = 145). For all our study participants, we did not find any current records of CD4 cell count. Few participants had baseline CD4 data (when they were being enrolled to the clinic). Therefore, logistic regression analyses did not include CD4 cell count information.

In the univariate logistic regression analyses, increasing perceived HIV-related stigma, being separated or divorced or widowed, on second-line cART regimen, having any current chronic illness or opportunistic infection and clinic inaccessibility were significantly associated with an increased risk for positive depression screen (*p < 0.05*; Table 1). Having no education or secondary education (relative to tertiary), living with a relative/friend (relative to family), and increased duration since HIV diagnosis were all reasonably associated with a positive depression screen at *p ≤ 0.20*. With increasing BMI, the risk of positive depression screen was 6% less likely at *p* = *0.11* (Table 1). All these variables were added to the multivariable logistic regression modelling in addition to age and sex (forced variables).

In the final multivariable analysis, factors that were significantly associated with positive depression screen among adults receiving cART in Kilifi included: having a current chronic illness (*p < 0.001*), being on second-line cART regimen (*p < 0.001*), clinic inaccessibility (*p = 0.02*), and increasing perceived HIV-related stigma (*p < 0.001*) (Table 3). The cross-validated mean area under the curve (cvMean AUC) for the final multivariable model was 0.75 (bootstrap bias corrected 95% CI: 0.66, 0.80).

**Table 3:** Multivariable logistic regression analysis for correlates of depressive symptoms in adults living with HIV from coastal Kenya

	Multivariable logistic regression analysis	
Predictor variable	aOR (95% CI)	*p*-value
Age	0.97 (0.94, 1.00)	0.07
Sex		
Female	Ref	
Male	0.72 (0.33, 1.57)	0.41
Any current chronic illness		
*No*	Ref	
*Yes*	5.72 (2.28, 14.34)	< 0.001
cART regimen		
*First line*	Ref	
*Second line*	6.93 (2.34, 20.49)	< 0.001
Clinic accessibility		
*Yes*	Ref	
*No*	2.37 (1.14, 4.91)	0.02
Perceived HIV-stigma	1.10 (1.05, 1.14)	< 0.001
BMI	0.94 (0.88, 1.01)	0.07
n of the final model	445	
Goodness of fit statistic	*p* = 0.79	
Variance explained by the model	pseudo-R^2^ = 16.1%	

Notes: *aOR* adjusted odds ratio, 95% CI - 95% confidence interval, *Ref* reference category, *cART* combination antiretroviral therapy, *BMI* body mass index

## Discussion

We report a relatively high prevalence of depressive symptoms (*13.8, 95% CI: 10.9, 17.3*) among PLWHA on cART on the Kenyan coast. We found that the presence of any chronic illness, being on second-line cART treatment, perceived HIV-related stigma, and problems of accessing HIV-related services among our study participants were significant correlates of positive depression screen.

The reported prevalence is considerably higher than the national prevalence of depression in Kenya of 4.4% [1], but is similar to estimates (ranging between 11 to 16%, with their respective 95% confidence intervals around our estimate) reported from studies conducted among PLWHA in Ethiopia [20, 30, 31], Rwanda [32], and Uganda [33]. However, the prevalence estimate in our study was much lower than what is reported in other studies from SSA such as Endeshaw et al. [34] in Ethiopia (60%), Kitshoff and Naidoo [35] in South Africa (62%), L’akoa et al. [36] in Cameroon (63%), and Shittu et al. [37] in Nigeria (57%). This could be due to differences in sample size, study population (selection bias), or tools used across studies. Analysis of findings reported by Endeshaw et al. [34] only involved a sample size of 55 participants. Kitshoff and Naidoo [35] used the Center for Epidemiologic Studies -Depression Scale (CES-D) to screen for depressive symptoms. The study by L’akoa et al. [36] was on newly diagnosed HIV patients, who are twice as likely than the general population of PLWHA to be depressed [38]. Shittu et al. [37] recruited both cART-experienced and cART-naïve patients.

In primary health care of most low resourced settings, depression is under-diagnosed and under-treated [8, 26]. In Kenya, and by extension similar low-resourced settings of SSA, poor integration of mental health services in primary care [39], scarcity of specialized mental health personnel [40], and a lack of mental health knowledge among primary health care staff [40] constitute barriers to the adequate identification, treatment or referral of those with mental health issues. Such barriers may contribute to the existence of relatively high levels of depressive symptoms among PLWHA reported in our study, but also in other studies from SSA.

In trying to address the burden of depression among PLWHA in SSA, calls were made to integrate mental health services in routine HIV care [8, 41]. In Kenya, as of 2016, strides were made to develop guidelines [24] in which screening for depression is recommended as a core aspect of HIV care. Under the new guidelines, healthcare providers at the HIV clinics are required to assess for depressive symptoms using the PHQ-9. However, national implementation of such guidelines remains a key challenge, and this is perhaps the case in other similar settings. To successfully integrate mental health screening into HIV primary care in our setting and similar settings, we call for the training of healthcare providers at HIV clinics on detection, management of, or referral for common mental health problems. Early detection will facilitate early management or referral for specialized care, hence better outcomes.

It has been observed that a growing number of adults in their 40s and 50s with long-term HIV and treatment histories are experiencing concurrent chronic diseases related to premature aging [42], similar to the multi-morbidity common in elderly populations [43]. In this study, the mean age of participants was 42.7 years, and duration on cART averaged 82 months (about 7 years). Using this sample, participants living with HIV and comorbid chronic illness (one or more chronic illness in addition to HIV) had nearly 6-times higher odds of a positive depression screen than those without any chronic illness. Our finding resonates with findings from a study conducted in the United States (US) among PLWHA 18 years old or above [44]. Uebelacker et al. [44] found that participants with moderate-severe chronic pain had higher levels of depressive symptoms relative to those with no chronic pain (*OR 1.13, 95% CI: 1.07, 1.20*). In PLWHA, additional comorbid chronic illness may limit an individual’s mobility, independence or make it impossible for one to pursue activities they usually enjoy leading to signs of despair, sadness, and loss of hope in the future manifesting as depressive symptoms.

We asked our participants about accessibility of the HIV clinic they were currently attending, by asking them to consider the distance between where they lived and the current location of the clinic. Less than a quarter of the participants (14%) found the clinic inaccessible, and this was significantly associated with higher odds of a positive depression screen. We presume that participants who found the HIV clinic inaccessible live in remote areas of Kilifi County and must travel long distances to access HIV-related services. Costs of travelling back and forth among other factors may hinder regular clinic attendance. Over time, the cumulative psychological distress related to fear of poor health outcomes can exacerbate and manifest as depressive symptoms. Future studies, mapping exact distances and travel time, should explore this finding further as there are no other studies to compare our findings in SSA. In a prospective cohort study of persons newly diagnosed with HIV infection from the US, Bhatia et al. [38] found that depression was associated with baseline poor access to care.

Participants who were on second-line cART regimen relative to those on first-line regimen were nearly 7-times more likely to have depressive symptoms. A change from first-line to second-line is a major step and is usually indicated because of cART toxicity or first-line cART resistance (often due to adherence issues) [45]. Whichever the case, there is an underlying immunologic failure which may trigger depressive symptoms when a patient is informed.

Among PLWHA, previous studies within SSA [31, 46] and outside [47, 48] have consistently found that perceived HIV-related stigma is significantly associated with greater symptoms of depression. Similarly, we found that the risk of positive depression screen was 1.1-times higher among participants who reported increased perceived HIV-related stigma. HIV-related stigma may lead to poor engagement with care and ultimately, worse health outcomes [34]. Therefore, the need for comprehensive HIV/AIDS awareness and stigma reduction intervention programmes – using multiple channels and targeting entire communities – needs to be continually addressed.

This study has some limitations. The cross-sectional study design limits any causal inference. Because of the sampling approach and selection criterion, the study findings may not be generalizable to the adult population living with HIV who are not actively attending HIV-related services/care and the elderly people (above 60 years) living with HIV. We also used PHQ-9 as a depression screening tool. As such, we only report the prevalence of depressive symptoms and not clinical depression. Previous studies report social support, CD4 cell count, and viral load as significant correlates of depressive symptoms among PLWHA [4, 46]. However, we did not collect information on social support and data on CD4 cell count, and viral load were missing from clinical records (our primary data source for clinical information). Consequently, these were not included in our analyses. Lastly, interviewer administration of study instruments in a face-to-face format may have introduced social desirability bias when asking participants sensitive and/or personal questions, e.g. “over the last 2 weeks, how often have you been bothered by thoughts that you would be better off dead or of hurting yourself in some way” item of the PHQ-9.

By recruiting a relatively large sample size, this study was well powered to estimate the prevalence and investigate factors associated with depressive symptoms among PLWHA. Even though PHQ-9 was used as a depression screener, it has been found reliable for use with PLWHA in Kenya [22], and our psychometric results also support the reliability of this tool.

## Conclusion

This study found a high prevalence (14%) of moderate to severe depressive symptoms among adults receiving cART on the Kenyan coast. Integration of mental health screening into HIV primary care in our setting and similar settings is highly recommended to facilitate treatment or referral services. Where this has been initiated, we call for a timely nationwide implementation given the reported success of integration efforts in other low resourced settings [41, 49]. Identification should target PLWHA with comorbid chronic illnesses, those on second-line cART regimen, experiencing HIV-related stigma, having problems accessing HIV-related services among other high-risk groups for elevated depressive symptomatology.

## Data Availability

No additional data are available. Anyone interested in accessing the data reported in this article is free to write to the Data Governance Committee of the KEMRI Wellcome Trust Research Programme who will review the application and advise as appropriate, and ensure that uses are compatible with the consent obtained from participants for data collection. Requests can be sent to the coordinator of the Data Governance Committee using the following email: dgc@kemri-wellcome.org

## Abbreviations

cART: combination antiretroviral therapy
CD4: Cluster of differentiation 4
CES-D: Center for Epidemiologic Studies -Depression Scale
CGMR: Centre for Geographic Medicine
cvMean AUC: cross-validated mean area under the curve
ICC: Intra-class correlation coefficient
PHQ-9: 9 item Patient Health Questionnaire
PLWHA: People living with HIV/AIDS
SSA: Sub-Saharan Africa
US: United States
WHO: World Health Organization

## References

[1] World Health Organization (WHO). Depression and other common mental disorders: global health estimates. 2017; Available from: http://apps.who.int/iris/bitstream/10665/254610/1/WHO-MSD-MER-2017.2-eng.pdf?ua=1&ua=1.

[2] Mathers CD, Loncar D (2006). Projections of global mortality and burden of disease from 2002 to 2030. PLoS Med.

[3] Ciesla JA, Roberts JE (2001). Meta-analysis of the relationship between HIV infection and risk for depressive disorders. Am J Psychiatr.

[4] Bernard C, Dabis F, de Rekeneire N (2017). Prevalence and factors associated with depression in people living with HIV in sub-Saharan Africa: a systematic review and meta-analysis. PLoS One.

[5] Joint United Nations Programme on HIV/AIDS (UNAIDS). Global HIV & AIDS statistics — 2018 fact sheet. 2018; Available from: http://www.unaids.org/sites/default/files/media_asset/UNAIDS_FactSheet_en.pdf.

[6] Ng’ang’a PW (2018). Undetected psychiatric morbidity among HIV/AIDS patients attending comprehensive care clinic (CCC) in Nairobi Kenya: towards an integrated mental health care. Ann General Psychiatry.

[7] Secor AM (2015). Depression, substance abuse and stigma among men who have sex with men in coastal Kenya. AIDS (London, England).

[8] Abas M (2014). Depression in people living with HIV in sub-Saharan Africa: time to act. Tropical Med Int Health.

[9] Uthman OA (2014). Depression and adherence to antiretroviral therapy in low-, middle-and high-income countries: a systematic review and meta-analysis. Current HIV/AIDS Reports.

[10] Nyongesa MK (2018). Neurocognitive and mental health outcomes and association with quality of life among adults living with HIV: a cross-sectional focus on a low-literacy population from coastal Kenya. BMJ Open.

[11] Kingori C, Haile ZT, Ngatia P (2015). Depression symptoms, social support and overall health among HIV-positive individuals in Kenya. Int J STD AIDS.

[12] Kinyanda E (2012). The prevalence and characteristics of suicidality in HIV/AIDS as seen in an African population in Entebbe district, Uganda. BMC Psychiatry.

[13] Amare T (2018). Prevalence and associated factors of depression among PLHIV in Ethiopia: systematic review and meta-analysis, 2017. AIDS Res Treat.

[14] Seth P (2014). Psychosocial functioning and depressive symptoms among HIV-positive persons receiving care and treatment in Kenya, Namibia, and Tanzania. Prev Sci.

[15] Korhonen C (2018). Depressive symptoms and problematic alcohol and other substance use in 1476 gay, bisexual, and other MSM at three research sites in Kenya. AIDS (London, England).

[16] Scott JAG (2012). Profile: the Kilifi health and demographic surveillance system (KHDSS). Int J Epidemiol.

[17] Commission on Revenue Allocation (CRA). Kenya County Fact sheets. 2011; Available from: http://siteresources.worldbank.org/INTAFRICA/Resources/257994-1335471959878/Kenya_County_Fact_Sheets_Dec2011.pdf.

[18] National AIDS Control Council (NACC). Kenya HIV County Profiles *-* 2016. 2016; 49–52]. Available from: http://nacc.or.ke/wp-content/uploads/2016/12/Kenya-HIV-County-Profiles-2016.pdf.

[19] Hunger M (2011). Multimorbidity and health-related quality of life in the older population: results from the German KORA-age study. Health Qual Life Outcomes.

[20] Yakob B, Purity Ncama B (2016). Client satisfaction: correlates and implications for improving HIV/AIDS treatment and care services in southern Ethiopia. Int Health.

[21] Kroenke K, Spitzer RL, Williams JB (2001). The PHQ-9: validity of a brief depression severity measure. J Gen Intern Med.

[22] Monahan PO (2009). Validity/reliability of PHQ-9 and PHQ-2 depression scales among adults living with HIV/AIDS in western Kenya. J Gen Intern Med.

[23] Akena D (2013). Sensitivity and specificity of clinician administered screening instruments in detecting depression among HIV-positive individuals in Uganda. AIDS Care.

[24] Ministry of Health (MoH). Guidelines on Use of Antiretroviral Drugs for Treating and Preventing HIV Infection in Kenya. 2016; Available from: https://aidsfree.usaid.gov/sites/default/files/kenya_art_2016.pdf.

[25] Cholera R (2014). Validity of the patient health questionnaire-9 to screen for depression in a high-HIV burden primary healthcare clinic in Johannesburg, South Africa. J Affect Disord.

[26] Gelaye B (2013). Validity of the patient health questionnaire-9 for depression screening and diagnosis in East Africa. Psychiatry Res.

[27] Reinius M (2017). Development of a 12-item short version of the HIV stigma scale. Health Qual Life Outcomes.

[28] Bursac Z (2008). Purposeful selection of variables in logistic regression. Source Code Biol Med.

[29] StataCorp LP (2015). Stata statistical software: release 14.

[30] Alemu H (2012). Effect of depressive symptoms and social support on weight and CD4 count increase at HIV clinic in Ethiopia. AIDS Care.

[31] Kibret GD, Salilih SZ (2015). Prevalence and associated factors of depression among HIV infected patients in Debre Markos town Northwest Ethiopia. Int J Emerg Ment Health Hum Resilience.

[32] Wroe EB (2015). Depression and patterns of self-reported adherence to antiretroviral therapy in Rwanda. Int J STD AIDS.

[33] Wagner GJ (2011). Understanding the influence of depression on self-efficacy, work status and condom use among HIV clients in Uganda. J Psychosom Res.

[34] Endeshaw M (2014). Stigma in Ethiopia: association with depressive symptoms in people with HIV. AIDS Care.

[35] Kitshoff C, Naidoo S. The association between depression and adherence to antiretroviral therapy in HIV-positive patients, KwaZulu-Natal, South Africa. S Afr Fam Pract. 2012;54(2):145–50.

[36] L’akoa RM (2013). Prevalence and correlates of depressive symptoms in HIV-positive patients: a cross-sectional study among newly diagnosed patients in Yaoundé, Cameroon. BMC psychiatry.

[37] Shittu R (2013). Prevalence and correlates of depressive disorders among people living with HIV/AIDS. North Central Nigeria. J AIDS Clin Res.

[38] Bhatia R (2011). Persons newly diagnosed with HIV infection are at high risk for depression and poor linkage to care: results from the steps study. AIDS Behav.

[39] Jenkins R (2010). Integration of mental health into primary care and community health working in Kenya: context, rationale, coverage and sustainability. Ment Health Fam Med.

[40] Kiima D, Jenkins R (2010). Mental health policy in Kenya-an integrated approach to scaling up equitable care for poor populations. Int J Ment Heal Syst.

[41] Akena D, Stein DJ, Joska J (2013). Does screening HIV-positive individuals in Uganda for major depressive disorder improve case detection rates and antidepressant prescription?. AIDS Behav.

[42] Balderson BH (2013). Chronic illness burden and quality of life in an aging HIV population. AIDS Care.

[43] Lee PG, Cigolle C, Blaum C (2009). The co-occurrence of chronic diseases and geriatric syndromes: the health and retirement study. J Am Geriatr Soc.

[44] Uebelacker LA (2015). Chronic pain in HIV-infected patients: relationship to depression, substance use, and mental health and pain treatment. Pain Med (Malden, Mass.).

[45] Meintjes G (2017). Adult antiretroviral therapy guidelines 2017. Southern African J HIV Med.

[46] Tesfaw G (2016). Prevalence and correlates of depression and anxiety among patients with HIV on-follow up at alert hospital, Addis Ababa, Ethiopia. BMC psychiatry.

[47] Charles B (2012). Association between stigma, depression and quality of life of people living with HIV/AIDS (PLHA) in South India – a community based cross sectional study. BMC Public Health.

[48] Yi S (2015). AIDS-related stigma and mental disorders among people living with HIV: a cross-sectional study in Cambodia. PLoS One.

[49] Udedi M (2018). Integrating depression management into HIV primary care in Central Malawi: the implementation of a pilot capacity building program. BMC Health Serv Res.

